# Supplementary material for: Individual and community level determinants of short birth interval in Ethiopia: A multilevel analysis
Source: PLoS One. 2020 Jan 14;15(1):e0227798. doi: 10.1371/journal.pone.0227798 (PMC6959604; doi:10.1371/journal.pone.0227798)
Supplement: S1 Table — (DOCX) [file pone.0227798.s001.docx]

**Table 1: Description of variables used in the analysis**

| **Variables** | **Description and coding** |
| --- | --- |
| **Individual-level variables** | |
| Maternal age at first marriage (Age in 5-year groups) | 1=<19; 2=20-24; 3=25-29; 4=30+ |
| Maternal age at birth of the preceding child (Age in 5-year groups) | 1=<19; 2=20-24; 3=25-29; 4=30-34; 5=35+ |
| Marital status | Categorical variable indicating marital status of the respondents  (1= Separated/Divorced/Widowed; 2=Married) |
| Polygyny status | Categorical variable indicating the presence of more than one wife for respondents’ husband/partner  (1=Yes; 2=No; 3=Don’t know) |
| Maternal education level | Categorical variable indicating the highest educational level that respondents completed  (1=No Education; 2= Primary; 3=Secondary; 4=Higher) |
| Husband’s/partner’s education level | Categorical variable indicating the highest educational level completed by respondent’s Husband/partner  (1=No Education; 2= Primary; 3=Secondary; 4=Higher; 5=Don’t know) |
| Maternal occupation status | Categorical variable indicating whether the women were working or not.  (1=Not working; 2=Working) |
| Husband’s/partner’s occupation | Categorical variable indicating whether the husbands/partners were working or not.  (1=Not working; 2=Working; 3=Don’t know) |
| Wealth index | The wealth index provided with the dataset was used. DHS program provides a composite index of household amenities based on the principal component analysis (PCA) and classified the population into quintiles: (1st quintile (Poorest); 2^nd^ quintile; 3^rd^ quintile; 4^th^ quintile and 5^th^ quintile (Richest). A quintile is used as a measure of its relative socioeconomic level.  (1=Poorest; 2=Poorer; 3=Middle; 4=Richer; 5=Richest) |
| Sex of preceding child | Whether the child was male or female.  (1=Male; 2=Female) |
| Total number of children born before the index child | The total number of children born before the index child (considered as a continuous variable after checking the linearity assumption). |
| Survival of preceding child | Whether the immediately preceding child was survived or dead  (1=Yes; 2=No) |
| Watched television | Whether the women watched television or not  (1=Yes; 2=No) |
| Listened to radio | Whether the women listened to the radio or not.  (1=Yes; 2=No) |
| Read newspapers | Whether the women read a newspaper or not.  (1=Yes; 2=No) |
| Distance to the health facility | Whether the women perceived the distance to the health facility to get medical help is a big problem or not.  1=Big problem; 2=Not big problem |
| **Community-level variable** | |
| Place of residence | Whether the cluster was an urban community, according to the definition of the country categorized as 1 and if the cluster was a rural community then categorized as 2.  (1=Urban; 2=Rural) |
| Contextual regions | Based on their living ways, the Ministry of Health of Ethiopia has classified regions into agrarian, pastoralist or city dwellers so as to make a contextual intervention for each region. Detail explanation about the contextual region can be found in the FMoH document (28).  (1=Agrarian (Tigray, Amhara, Oromia, SNNPR);  2=Pastoralist (Afar, Somali, Benishangul-Gumuz & Gambela); 3=Cities dweller (Addis Ababa & Dire Dawa)) |
| Community-level female literacy | It is dichotomous variables indicating whether the proportion of women aged 15-49 in the community with secondary or higher education was high or low (cut-off at median proportion).  (1=High; 2=Low) |
| Community-level poverty | The proportion of poor households (two lowest wealth quintiles) in the community (cut-off at median proportion).  (1=High; 2=Low) |
| Community-level distance to the health facilities as a big problem | The proportion of women aged 15-49 in the community who perceive the distance to the health facilities to get medical help was a big problem (cut-off at median proportion).  (1=High; 1= Low) |

SNNPR=Southern Nations, Nationalities and Peoples’ Region
