# Supplementary material for: Individual and community level determinants of short birth interval in Ethiopia: A multilevel analysis
Source: PLoS One. 2020 Jan 14;15(1):e0227798. doi: 10.1371/journal.pone.0227798 (PMC6959604; doi:10.1371/journal.pone.0227798)
Supplement: S2 Table — (DOCX) [file pone.0227798.s002.docx]

**Table 2: The results of multicollinearity analysis**

| **Variables** | **VIF** | **Tolerance =1/VIF** |
| --- | --- | --- |
| Maternal age at first marriage | 1.10 | 0.908766 |
| Maternal age at birth of the preceding child | 1.27 | 0.785738 |
| Polygyny status | 1.07 | 0.937206 |
| Maternal education level | 1.25 | 0.801318 |
| Husband’s/partner’s education level | 1.30 | 0.770782 |
| Maternal occupation status | 1.05 | 0.952075 |
| Husband’s/partner’s occupation | 1.06 | 0.947132 |
| Wealth index | 1.76 | 0.568368 |
| Sex of preceding child | 1.00 | 0.997302 |
| Total number of children born before the index child | 1.35 | 0.743045 |
| Survival of preceding child | 1.01 | 0.989403 |
| Watched television | 1.71 | 0.584397 |
| Listened to radio | 1.33 | 0.753450 |
| Read newspapers | 1.23 | 0.810588 |
| Distance to the health facility | 1.53 | 0.654700 |
| Place of residence | 1.80 | 0.554306 |
| Contextual regions | 1.38 | 0.726401 |
| Community-level female illiteracy | 1.43 | 0.697408 |
| Community-level poverty | 1.98 | 0.505539 |
| Community-level distance to the health facilities as a big problem | 1.64 | 0.609343 |

VIF=Variance inflation factor
